# 正确认识病原微生物宏基因组二代测序技术在血液病感染病原诊断中的价值

**DOI:** 10.3760/cma.j.cn121090-20241029-00423

**Published:** 2024-11

**Authors:** 四洲 冯, 春晖 徐

**Affiliations:** 1 中国医学科学院血液病医院（中国医学科学院血液学研究所），血液与健康全国重点实验室，国家血液系统疾病临床医学研究中心，细胞生态海河实验室，天津 300020 State Key Laboratory of Experimental Hematology, National Clinical Research Center for Blood Diseases, Haihe Laboratory of Cell Ecosystem, Institute of Hematology & Blood Diseases Hospital, Chinese Academy of Medical Sciences & Peking Union Medical College, Tianjin 300020, China; 2 天津医学健康研究院，天津 301600 Tianjin Institutes of Health Science, Tianjin 301600, China

## Abstract

血液病患者易发感染，且感染相关死亡率较高，感染病原的准确诊断至关重要，但常规微生物检测难以满足临床需求，成为临床面临的重要难题。宏基因组二代测序（mNGS）因其高灵敏度、广覆盖及检测周期短的特点，已在感染性疾病诊断中获得广泛应用。本文聚焦mNGS技术在血液病患者感染病原诊断中的应用现状，分析其在临床中面临的挑战和未来发展趋势，旨在帮助临床医师深入认识该技术在血液病感染诊断中的价值及局限性，以实现合理应用。

## Abstract (en)

Patients with hematologic diseases are prone to infections, and infection-related mortality is high in this population. Accurate diagnosis of infectious pathogens is crucial; however, conventional microbiological testing often fails to meet clinical needs, presenting a significant challenge in clinical practice. Metagenomic next-generation sequencing（mNGS）, with its high sensitivity, broad coverage, and short turnaround time, has been widely adopted in diagnosing infectious diseases. This article focuses on the application of mNGS technology in diagnosing infectious pathogens in patients with hematologic diseases. It analyzes the challenges encountered in clinical practice and explores future trends in the field. This work aims to provide clinicians with an in-depth understanding of the value and limitations of mNGS in diagnosing infections in hematologic patients, and to promote its rational application.

血液病患者由于疾病本身或治疗过程中免疫系统功能受到抑制，容易伴发感染，感染灶往往隐匿，感染特征不明显，病原诊断困难[1]。传统微生物学检测周期长，检测谱相对有限，且部分血液病患者感染在病原微生物检测前已经接受了经验性抗感染药物治疗，导致病原微生物培养阳性率进一步降低，难以获得病原学诊断证据。随着分子生物学技术的快速发展，病原微生物宏基因组二代测序（metagenomic next-generation sequencing，mNGS）作为一种覆盖广、灵敏度高、报告周期短的病原检测工具，已广泛应用于临床感染性疾病病原诊断，多项研究表明mNGS技术对指导血液病患者的感染诊治具有显著优势，明显提高了感染病原检出率[2]–[8]。mNGS技术在血液病感染病原诊断中的应用潜力巨大，也面临着许多挑战和局限。

一、mNGS技术在血液病感染病原诊断中的临床应用

在血液病患者人群中，近年来已有多篇mNGS应用于血流感染、粒细胞缺乏（粒缺）伴发热、中枢神经系统感染以及呼吸道感染病原诊断的研究论文相继发表。mNGS相对于传统微生物学检测具有如下优势：（1）阳性率高，检测周期短，受到抗菌药物的影响小于传统微生物学检测。国内一项多中心前瞻性研究显示，在细菌和真菌的检出方面，血液样本采集前1 d内抗菌药物使用可以显著降低血培养阳性率（23.8%对13.5%，*P*＝0.016），而对mNGS检测的阳性率几乎无影响（46.2%对51.9%，*P*＝0.278）[2]。目前mNGS检测周期多在24 h以内，相比培养方式可有效缩短检测周期。（2）对于混合感染具有明显的检测优势。（3）对于少见病原或传统微生物检测未覆盖的病原，具有早期诊断能力。近年来众多mNGS研究报道了多种传统检测中罕见检出的病原种类，例如细菌中的非结核分枝杆菌、军团菌属内的非嗜肺军团菌种、真菌中的灰盖鬼伞霉、木霉以及寄生虫等。（4）血液样本可在局灶感染时作为替代选择，尤其在感染部位样本不可获取的情况下；基于游离核酸检测原理，外周血液标本对局灶感染（例如肺感染）具有一定检测能力，但值得注意的是，与感染部位样本相比，其检测性能仍有较大差距，可发生病原漏检。选择感染部位样本进行检测仍是最佳方案，应积极创造条件获取感染部位样本，尤其对于肺部感染，积极获取下呼吸道样本［如支气管肺泡灌洗液（BALF）］仍然是提高呼吸道感染病原诊断水平的关键所在。

在血液病患者感染病原诊断中，当可获得感染部位样本时，mNGS可以全面、高灵敏度地检出感染病原。有研究显示，在中枢神经系统感染中，脑脊液mNGS灵敏度可达97.1%；在呼吸道感染中，BALF mNGS的灵敏度可达81.0%～88.9%[5]–[7]。在血液病患者的感染过程中，由于血小板低以及重度免疫抑制，与其他学科（如呼吸科、重症科、急诊科等）患者相比，感染部位样本的获取更为困难，因此血液样本也是常见的检测类型。已有研究将血液样本mNGS应用于呼吸道感染、皮肤软组织感染、腹腔感染以及特殊病原（如少见细菌、真菌、寄生虫）感染中，在病原和（或）核酸入血的情况下检出微生物。其中毛霉菌病是血液病患者并非少见的真菌感染类型，由于其培养阳性率低且常见生物标志物为阴性，使得其早期诊断非常困难。由于该类真菌具有噬血管性，且粒缺患者血液样本检测敏感性明显高于非粒缺患者，血液样本mNGS一旦检出，往往提示存在侵袭性毛霉菌病[4],[8]。有研究显示，侵袭性肺真菌病发生时，尽管肺组织检测与BALF的灵敏度无明显差异，但组织样本的序列数通常高于BALF。此外，在毛霉病感染时，可出现组织样本检出病原而BALF未检出的情况。这一现象通常与丝状真菌的侵袭性有关，灌洗法往往很难冲掉病原体[9]。

尽管mNGS检测较高阳性率在一定程度上解决了血液病感染病原诊断的难题，但在临床应用中仍存在一些需要关注的问题：（1）mNGS不能完全取代传统微生物学检测。首先，尽管与传统病原菌培养相比，mNGS阳性率显著增高，但仍然存在漏检可能；无论是由于样本中人源核酸比例过高导致的病原检测性能减弱，还是部分病原破壁困难导致诸如真菌、分枝杆菌的漏检，分子检测并不能完全取代培养、镜检及生物标志物等传统微生物检测方式；此外，mNGS检测尚不能作为感染确诊或临床诊断的标准，其检出的病原需经传统微生物学检测验证，尤其是对于低序列检出的病原，更应结合传统微生物学检测结果进行综合判断。（2）样本质量控制与恰当采集至关重要。检测结果与样本质量密切相关，应建立严格的样本质量控制标准，对不合格样本应重新送检。例如，严重溶血的样本会显著降低病原检出灵敏度，而不合格的痰液样本往往只能检出呼吸道定植菌，如能在影像技术支持下，从感染部位采集BALF回收液和（或）组织样本则可提高病原检出率[9]。（3）mNGS检测结果需要临床医师谨慎解读。医师需充分结合患者感染的临床特点及微生物致病性判断是否为致病病原。对于致病性强的微生物如结核分枝杆菌、支原体、衣原体、隐球菌及毛霉目真菌，即使序列低，也应充分考虑是致病病原的可能，尤其对于隐球菌和结核分枝杆菌，由于其细胞壁厚，导致破壁困难，通常呈现较低的检出序列。此外，样本mNGS病毒检出常见，其临床意义与病毒种类、患者免疫状态高度相关。对于造血干细胞移植后患者，如出现中枢神经系统感染，脑脊液中检出HHV-6B应高度考虑为致病病原，而对于CMV、EBV这类可潜伏在细胞内的病毒，其检出并非一定是致病病原，若有怀疑，应该通过其他方法验证。对于血液样本，若CMV、EBV的PCR定量检测阴性，仅mNGS中检出，则CMV、EBV是感染病原的可能性极小。此外，细环病毒、庚型肝炎病毒因其并无循证学依据导致明确的感染，其检出不应考虑为患者感染病原。（4）应对mNGS检测的适应证进行规范。尽管近年来高通量测序成本有所减低，但目前临床可及的mNGS检测价格仍远高于传统微生物学检测，2023年发布的《宏基因组二代测序技术在血液病患者感染病原诊断中的应用中国专家共识（2023年版）》对推荐送检时机进行了规范，其适应证仍应以“急、危、重、难”为主[8]。

二、病原mNGS技术应用存在的挑战

mNGS检测作为一项高通量测序技术，其检测流程包括湿试验与干试验，但在检测环节仍存在诸多尚未解决的问题。首先，污染仍是mNGS检测的常见问题，其“假阳性”微生物既可通过湿试验引入，也可由生信环节产生[10]；湿试验的污染主要来自试剂或实验室环境中的微生物，当污染微生物也是潜在感染病原时，结果的分析和解释则变得复杂和难以判断。此外，样本采集过程中，定植微生物的干扰也会在一定程度上影响报告的解读。例如在BALF的采集过程中难免受到口腔菌干扰，而这些微生物在一定情况下（如吸入性肺炎）也可成为致病病原；因此，严格把控试验流程与质控体系，定期的背景菌分析对于实验检测质量尤为重要；同时，对既往罕见报道的微生物检出进行重复验证，也是减少假阳性的重要举措之一。其次，“人源核酸”作为检测中未被分析的部分，直接影响微生物检测灵敏度。当样本中人源序列占比过高时，微生物占比相对减小，在固定数据量的情况下，微生物检出的灵敏度随之下降。尽管目前可通过差异裂解、核酸片段大小筛选等方式进行试验流程的“去人源”，但如何判断样本是否需要“去人源”处理以及如何恰当地进行“去人源”处理仍然存在挑战。此外，mNGS结果的准确性依赖于微生物数据库的分析，数据库中信息的准确性和全面性是准确报告微生物的前提条件。部分微生物数据库信息尚不完备，可能导致微生物的漏报、错报。最后，在耐药与毒力基因分析上，mNGS由于其短读长的特点（通常为50 bp），难以对基因进行准确定位，基因型与表型是否匹配应进行充分验证[11]。

近年来，靶向二代测序（target NGS, tNGS）也逐渐应用于临床感染病原诊断。与mNGS相比，tNGS在测序前通过PCR或探针捕获的方式富集微生物核酸，从而显著降低测序成本。目前，tNGS已在呼吸道感染病原诊断中得到较为广泛的应用，并有多项研究对其性能及临床价值进行了评估[12]–[14]。目前尚未有针对血液病患者群体的研究报道。值得注意的是，tNGS的检测病原范畴相对固定，检测前应对可检测范畴进行充分分析，判断是否满足检测人群及本地域感染病原流行病学的检测需求。近期北京大学人民医院王辉教授团队发表的基于呼吸道样本mNGS与两种tNGS检测平行比较的研究显示，tNGS在少见丝状真菌检测性能上略弱于mNGS检测（如毛霉目），而该类真菌是血液病患者并非少见感染病原类型。此外，基于靶向扩增的模式，tNGS检出序列与病原真实存在构成也有较大的差异，其检测流程标准化、病原谱设计和报告解读均有待进一步优化与完善。

三、未来展望

随着病原mNGS技术的不断发展，未来其在血液病患者感染病原检测中的应用前景令人期待。

首先，mNGS技术在灵敏度和特异性方面的优势将持续推动其在感染病原诊断中应用普及。传统微生物检测方法常受限于病原体的培养条件和检测时间，而mNGS通过全面的基因组测序，能够快速识别多种病原体，特别是在复杂的多重感染情况下，为临床医师提供了更可靠的病原信息。随着技术的进一步优化和数据分析能力的提升，mNGS有望实现更短的检测周期和更低的检测成本，进而提高检测的普及和普适性，帮助临床医师及时做出决策，从而改善血液病患者感染预后。

其次，mNGS技术将在不断迭代中实现更有针对性的靶向检测，有望在血液病感染领域发挥更大作用。在tNGS检测方面，随着技术的不断优化，tNGS可检测样本类型将得到扩展，结合不同病种的感染特征及地域化感染流行病学信息，有望更好地服务于临床，实现针对不同感染类型的精准检测。此外，在mNGS检测领域，目前大量的人源信息尚未被充分应用。已有多项研究利用人源信息辅助肿瘤识别、移植物抗宿主病判断、炎症反应分析[15]–[16]，未来通过微生物数据信息结合人源信息综合判断有望加强临床精准诊断。

最后，mNGS技术的未来展望还包括其在公共卫生和流行病学监测中的应用。随着新型病原体识别和监测能力的增强，mNGS检测可以为流行病学研究提供重要的数据支持，帮助公共卫生部门及时响应潜在的感染暴发并有效控制院内感染。在血液病患者中，早期识别病原和提高预防感染的能力对于降低感染并发症发生率和改善血液病患者生存率至关重要。未来，结合人工智能与机器学习等新兴技术，可以实现更智能化、更大数据量的分析与预测，为血液病患者感染诊治提供精准服务。
